# The Modelling of Hand, Foot, and Mouth Disease in Contaminated Environments in Bangkok, Thailand

**DOI:** 10.1155/2018/5168931

**Published:** 2018-06-03

**Authors:** Sudarat Chadsuthi, Surapa Wichapeng

**Affiliations:** ^1^Department of Physics, Research Center for Academic Excellence in Applied Physics, Faculty of Science, Naresuan University, Phitsanulok 65000, Thailand; ^2^Department of Physics, Faculty of Science, Naresuan University, Phitsanulok 65000, Thailand

## Abstract

Hand, foot, and mouth disease (HFMD) has spread widely in a continuing endemic in Thailand. There are no specific vaccines or antiviral treatments available that specifically target HFMD. Indirect transmission via free-living viruses from the environment may influence HFMD infections because the virus can survive for long periods in the environment. In this study, a new mathematical model is proposed to investigate the effect of indirect transmission from contaminated environments and the impact of asymptomatic individuals. By fitting our model to reported data on hospitalized individuals of HFMD endemic in Bangkok, Thailand, 2016, the basic reproduction number was estimated as 1.441, which suggests that the disease will remain under current conditions. Numerical simulations show that the direct transmission from asymptomatic individuals and indirect transmission via free-living viruses are important factors which contribute to new HFMD infections. Sensitivity analysis indicates that the basic reproduction number is sensitive to the transmission rate of asymptomatic and symptomatic subgroups and indirect transmission. Our findings suggest that cleaning the environment frequently and healthcare precautions which include the reduction of direct transmission rates should be promoted as effective control strategies for preventing the HFMD spread.

## 1. Introduction

Hand, foot, and mouth disease (HFMD) is an emerging illness, which most frequently caused by coxsackievirus A16 (CA16) and enterovirus 71 (EV-71) infecting infants and children [1]. Symptoms of HFMD are fever, painful sores in the mouth, and a rash with blisters on hands, feet, and also buttocks [2]. HFMD spreads mainly in children, who are under five years old, because they are more likely to be susceptible to infection by these viruses [2]. Most adults have antibodies and are immune due to previous exposures leading to less chance of infection [2].

Since the 1970s, the outbreaks of HFMD have been reported. The disease has spread in many countries of the western Pacific region, which is the most severely affected region in the world, such as Japan [3], Malaysia [4], Singapore [5], Thailand [6], and China [7]. In Thailand since 2001, HFMD has been monitored under the National Disease Surveillance (report 506), Bureau of Epidemiology, Department of Disease Control, Ministry of Public Health, Thailand. At that time, a total of 1,548 cases were reported with the most cases being found in Bangkok which was estimated to be 70.74% of total cases [8]. The epidemic of HFMD has been continuing with an average annual incidence rate of about 85.78 cases per 100,000 population per year during the last five years [8]. The disease spread across Thailand, but mainly in Bangkok (average cases about 13% of total). Thus, the impact of HFMD has become a public health concern that includes social and economic problems.

The transmission of HFMD can occur from close contact with infected people, touching virus carrying objects, and contaminated water and food. In the environment, the pathogen EV-71 can survive for a long period in suitable conditions outside the host [9]. Also 75% of alcohol based cleaning products cannot eliminate the virus, while 95% ethanol is most effective but cannot fully deactivate pathogen EV-71 [10]. Hence, the disease can be transmitted through contact with contaminated environments such as water, food, or surfaces [11]. Most infected patients can recover in 7 to 10 days without medical treatment. However, the patients, who are infected by EV-71, have been associated with meningitis and encephalitis, and on occasion, it can cause severe complications, including neurological, cardiovascular, and respiratory problems [2]. The delay in diagnosis and treatment may cause severe clinical symptoms which can result in the death of infected children. Presently, there are no specific vaccines or antiviral treatments available for dealing with HFMD [2, 12].

Epidemiological models have been become important tools for investigating and understanding transmission dynamics and disease controls. A simple deterministic SIR model was used to predict the number of infected people and the duration of an outbreak when it occurred in Sarawak, Malaysia, by [13]. Later on, Liu proposed a periodic epidemic model and considered the effects of quarantine upon the child population [14]. Results show that the quarantine strategy is beneficial to the intervention and control of the disease outbreak. Li et al. applied the model without hospitalized but infectious and hospitalized infectious compartments to fit with HFMD data from China [15]. The roles of asymptomatic individuals and contaminated environments were investigated, and the numerical results show that both factors are important to delay and prevent epidemic outbreaks [16, 17]. However, the existing models are mostly considered to be limited to the epidemic in China. In Thailand, the HFMD models only analysed the effects of hand-washing to reduce the direct contact rate and school closure campaigns [18, 19]. The indirect transmission via free-living viruses from the environment and infectious individuals who do not present symptoms and individuals who present symptoms but are treated out of hospital and hospitalized individuals who present symptoms and have been admitted to hospital could affect the HFMD outbreak in Thailand.

The main purpose of this study is to extend the existing mathematical models by including the indirect transmission from contaminated environments [16, 17]. The model also considers subgroups of infected individuals to investigate the impact of asymptomatic individuals [20]. The numerous hospitalized individuals who are used to fit the HFMD reported data of Bangkok, Thailand. The basic reproduction number is provided. The simulations of the model and the sensitivity analysis regarding basic reproduction number are described.

## 2. Materials and Methods

### 2.1. Data

In this study, monthly cases of HFMD were retrieved from the National Disease Surveillance (report 506), Bureau of Epidemiology, Department of Disease Control, Ministry of Public Health, Thailand [8]. Most positive cases were suspected HFMD cases, based on clinical diagnosis made by attending physicians. The clinical criteria for HFMD were fever, spots and mouth ulcers, painful sores in the mouth, and a rash with blisters on hands, feet, and buttocks. Some suspected HFMD sample cases were then tested using viral culture for laboratory confirmation. The suspected HFMD cases are reported only from public hospitals and some private hospitals, although HFMD could spread in adults. However, most adults may have antibodies and are immune due to previous exposures [2]. During January–October 2012, the positive samples were found in mainly in children aged 5 years or younger [21]. In Thailand during 2013, the prevalence of enterovirus (EV) among patients with HFMD was found majorly in patients aged 2–6 years [22]. Moreover, monthly reported cases of HFMD in Bangkok 2016 (age < 12 years) were found more than 97% of total reported cases [8]. Thus, in this work, only HFMD cases, who are under the age of 12 (from birth to 12 years) in Bangkok 2016, were analysed. The number of reported cases in Bangkok exhibited more cases than other provinces in Thailand. The children of this group are in kindergarten and elementary school and have high chance of close contact with infected objects and people.

### 2.2. Model

**Figure 1:**
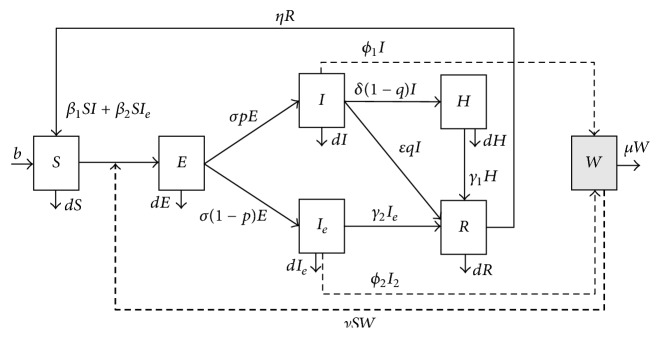
Flow chart of compartments of HFMD model.

To construct a model, we assumed that the population is homogeneous mixing and has an equal chance of contact with any individuals among the population. To simplify the model, we neglected the impacts of space, demographics, and season in analysing the data. The total population *N* in this model is only the group of children who is the target group of public health. The model includes direct transmission from infected individuals (both symptomatic and asymptomatic infected individuals) and indirect transmission from contaminated environments via free-living virus. The model compartment individuals are susceptible *S*, exposed *E* (exposed but not yet infectious), infectious but not presenting symptoms *I*_*e*_, infectious with symptoms *I*, hospitalized *H*, and recovered *R*. Let *W* represent the density of pathogens in contaminated environments including toys, door handles, tables, bedding, and underclothes [17]. A susceptible move to the exposed compartments was by infecting from direct contact via infected individuals or indirect contact via contaminated environments. After the latent period, these individuals become infectious (*I* or *I*_*e*_). Some of the infectious individuals (*I*) will be hospitalized for treatment and isolated from other individuals. The flow chart of the compartments of the HFMD model is shown in Figure 1.

In the environment in developing this model, we assumed that free-living viruses can live for weeks or months but they cannot reproduce by themselves. The rates at infectious individuals (*I* and *I*_*e*_) spread the virus in the environment being *ϕ*_1_ and *ϕ*_2_. The pathogens can be clear due to the sterilization at rate *μ*. The definition of all parameters is listed in Table 1. The set of model equations are as follows:

(1) dSdt=b−β1SI+β2SIeN−νSW+ηR−dSdEdt=β1SI+β2SIeN−σE+νSW−dEdIdt=σpE−δ1−qI−εqI−dIdIedt=σ1−pE−γ2Ie−dIedHdt=δ1−qI−γ1H−dHdRdt=εqI+γ2Ie+γ2H−ηR−dRdWdt=ϕ1I+ϕ2Ie−μW−νS+E+I+Ie+RW.

Note that the parameter *ν* is much less than the rate *μ*, which means that the loss of viruses due to individuals taking out is much less that the loss due to clearance. Hence, we can simplify the model by without considering the *ν*(*S* + *E* + *I* + *I*_*e*_ + *R*)*W* term in this study [16, 17].

The basic reproduction number (*R*_0_) of the system (1) is then calculated by using the next generation matrix approach in [23, 24] (see in Supplementary Materials for detail (available here)):

(2) $$\begin{array}{c} R_{0}=\frac{p\sigma \left(b\nu \phi_{1}+d\mu \beta_{1}\right)}{c_{1}c_{2}d\mu }+\frac{\left(1-p\right)\left(b\sigma \nu \phi_{2}+d\sigma \mu \beta_{2}\right)}{c_{1}c_{3}d\mu }, \end{array}$$

where *c*_1_ = *d* + *σ*, *c*_2_ = *d* + (1 − *q*)*δ* + *εq*, and *c*_3_ = *d* + *γ*_2_.

In epidemiology, the basic reproduction number (*R*_0_) is the average number of secondary infected individuals that are produced by a single infection in an entirely susceptible population. The first of this *R*_0_, *pσ*(*bνϕ*_1_ + *dμβ*_1_)/*c*_1_*c*_2_*dμ*, is the average number of secondary infected individuals that are generated by direct contact and indirect contact via contaminated environments (shed by symptomatic infected individuals) through a symptomatic infected individual. The second term (1 − *p*)(*bσνϕ*_2_ + *dσμβ*_2_)/*c*_1_*c*_3_*dμ* is the average number of secondary infected individuals, which are generated by direct and indirect contact through an asymptomatic infected individual (contaminated by free-living viruses in the environment).

### 2.3. Sensitivity Analysis

To investigate a sensitivity analysis on the basic reproduction number, *R*_0_, we used Latin Hypercube Sampling (LHS) and Partial Rank Correlation Coefficients (PRCC) techniques [25, 26]. LHS is a statistical Monte Carlo sampling, which is used to sample parameters that appear dependant on *R*_0_. Using “lhs” package version 0.14 in R software [27], 2000 parameter sets were sampled with LHS technique. In this study, we used uniform distributions for all dependant parameters [17], which are listed in Table 2. Based on the 2000 parameter sets, PRCC are computed using the “sensitivity” package version 1.15.10 in R software with bootstrapped 1,000 times to obtain 95% confidence intervals [28]. PRCC is used to measure which parameters have strongly linear associations on model outcomes, which are sensitivity indices based on linear assumptions, in the case of correlated factors [28].

## 3. Results

### 3.1. Parameter Estimation

In this work, we numerically analysed the system model (1). We assumed that the person's natural death rate is less compared to the rate of children who become adults. We only considered the population of children under twelve years old. The number of susceptible individuals in 2016 was obtained from National Statistical Office, Thailand, as *S*(0) about 716,297. We also obtained annual birth data from National Statistical Office, Thailand, and then calculated the birth population *b* = 252 each day. We assumed that the recovered individuals, who have immunity for HFMD, will lose their immunity and become susceptible individuals within 100 days [14].

**Figure 2:**
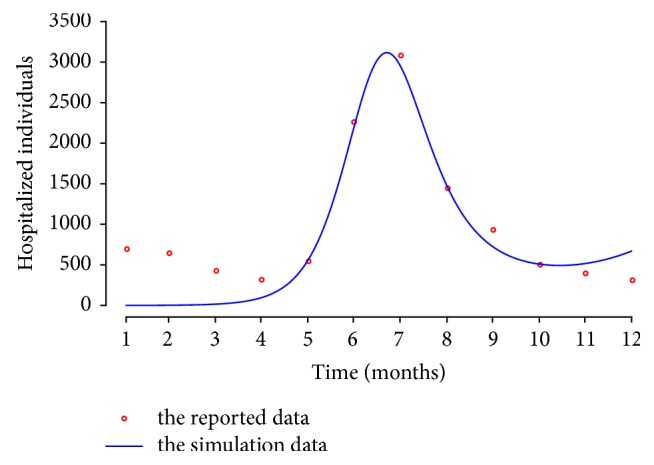
The comparison chart of the reported data of HFMD in Bangkok and simulation results.

By using a genetic optimization algorithm [29], we estimated the parameters of the system model (1), which was implemented using the “rgenoud” package version 5.7–1 for R software version 3.1.2 [30], which is a free software environment for statistical computing and graphics [31]. This algorithm can find the optimal parameter using biological approach of natural selection and mutation process. Each set of parameters can be randomly generated in each generation. Each set of parameters that are a candidate solution can evaluate fitness function and be sorted. A second pool of set of parameters is generated based on the previous generation and breeding them using the biological principles of mutation. Mutation allows passing to the next generation. The process will be repeated until the best parameters do not change. In this work, we set the population size at 500 and allowed for a maximum of 100 generations, which is satisfied to find the optimal parameters. We optimized the objective function as least square errors. We then fitted the model to the monthly reported data on HFMD cases.  Figure 2 shows the goodness of fit to the reported data in Bangkok 2016. By Pearson's chi-square test, the numerical results are found to be a good match with the reported data (*p* value < 0.001). The parameters are estimated as shown in Table 1.

### 3.2. Numerical Simulation and Sensitivity Analysis

By numerical simulation, we analysed the model focusing on the effect of asymptomatic infected individuals and indirect contact from contaminated environments. The basic reproduction number based on our parameter values was estimated as 1.441, which is more than a unit. This value can explain that HFMD is prevalent and still endemic in Bangkok. Thus, the result shows that the parameter estimation is close to reality. To study the impact of asymptomatic infected individuals, we let the transmission rate *β*_2_ equal to zero, then *R*_0_ is changed to 0.546. Similarly, if we neglect the impact of indirect transmission via contaminated environments (*ν* = 0), *R*_0_ becomes 1.028. Our result indicates that direct transmission from asymptomatic infected individuals and indirect transmission via free-living virus impact on *R*_0_, which contributes to the transmission dynamic of HFMD [16].

**Figure 3:**
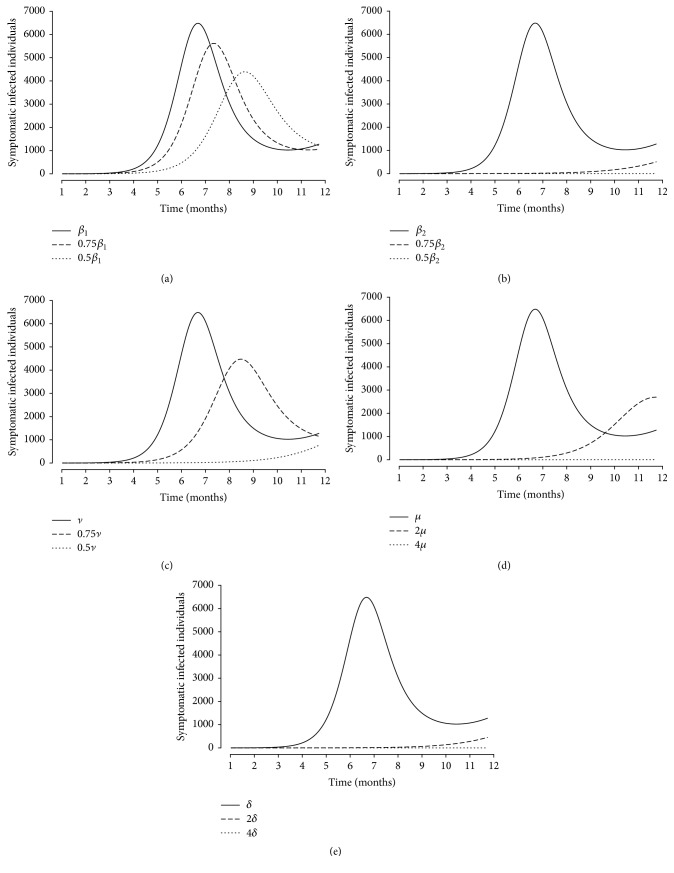
The number of symptomatic infected individuals (*I*(*t*) and *H*(*t*)) as (a) *β*_1_ is varied by 50% and 75% of its baseline value, (b) *β*_2_ is varied by 50% and 75% of its baseline value, (c) *ν* is varied by 50% and 75% of its baseline value, (d) *μ* is varied by 200% and 400% of its baseline value, and (e) *δ* is varied by 200% and 400% of its baseline value.

The results of reducing the transmission rate from symptomatic and asymptomatic individuals (Figures 3(a) and 3(b)) are compared to investigate the effect of asymptomatic individuals. It shows that the number of symptomatic infected individuals is affected by reducing *β*_2_ rather than by reducing *β*_1_. This result indicates that the disease transmission by the asymptomatic infected individuals could influence HFMD outbreak and should not be disregarded. The effect of contaminated environmental clearance can be investigated by reducing the indirect transmission rate (*ν*) and increasing the clearance pathogen rate (*μ*).  Figure 3(c) shows the number of symptomatic infected individuals reduced from the baseline value to 75% and 50% of *ν*. The increasing clearance pathogen rate (*μ*) is affected by reducing the number of symptomatic individuals (Figure 3(d)). This result implies that hygienic precautions and environmental clearance, such as asymptomatic individuals washing their hands and contact protection from contaminated environments, play important roles in reducing the number of infectious individuals and contributes to the effective control strategy, which could delay an HFMD outbreak. We further investigated the rate of symptomatic infected individual's progression to hospitalization.  Figure 3(e) shows that the number of symptomatic infected individuals decreases as this rate increases. It implies that symptomatic individuals should be hospitalized in time when they appear to have symptoms, thus reducing the chance of contact with other people as isolated individuals.

**Figure 4:**
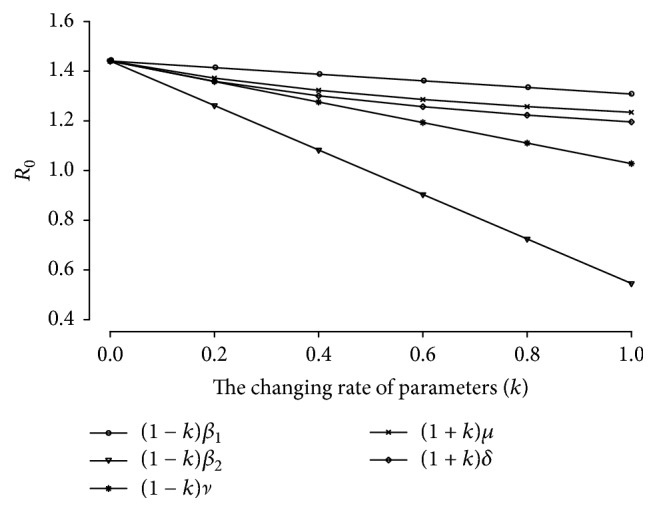
The basic reproduction number by varying *k*.

To compare the influence of those parameters on HFMD outbreak, we investigated the effects of varying *β*_1_, *β*_2_, *ν*, *μ*, and *δ* on the basic reproduction number [17].  Figure 4 shows the variation of the basic reproduction number by changing *k*. We found that decreasing the direct transmission by asymptomatic individuals is most effective. The second effective parameter reduces indirect transmission via free-living viruses in terms of reducing the reproduction number. When the changing rate *k* is equal to one, the interval of *R*_0_ of *β*_1_ and *β*_2_ is large. These may contribute to the impact of the number of asymptomatic individuals. The results suggest that we cannot disregard the contact rate of asymptomatic group. However, asymptomatic group could not be diagnosed or tracked. Consequently, it is impossible to achieve measure control. Our results also correspond to the results of [17]. Thus, this initiates healthcare concerns such as washing hands, cleaning and disinfecting toys, bedding, underclothes, and appliances, which should facilitate controlling HFMD infections.

**Figure 5:**
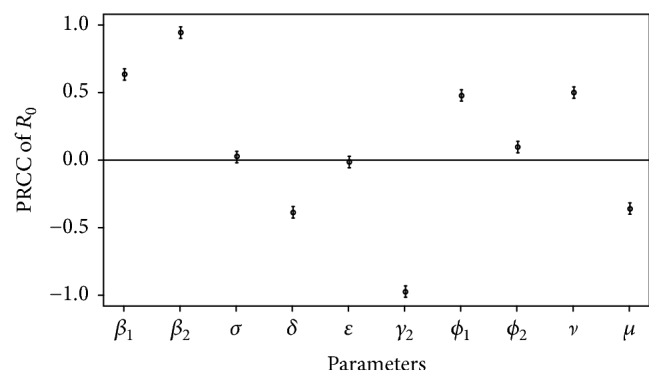
The values of PRCC on the outcome of *R*_0_. Bars represent 95% confidence intervals.

To determine the effect of each input parameter on the *R*_0_, we computed the PRCC values and standard error in Table 2 and plotted in Figure 5. We considered absolute values of PRCC > 0.3 as indicating important parameters for output variables. Figure 5 shows that the recovery rate of asymptomatic individuals (*γ*_2_) has the most impact on the basic reproduction number as followed by the direct transmission rate (*β*_2_ and *β*_1_), indirect transmission rate (*ν*), the shedding virus from symptoms individuals (*ϕ*_1_), the progression rate to hospital (*δ*), and the clearance pathogen rate (*μ*). Note that variations in the direct transmission rate (*β*_1_) have less effect on the number of symptomatic infected individuals (shown in Figure 3(a)) but highly correlated with average number of secondary infected individuals (determined by *R*_0_), while the variation of the progression rate to hospital (*δ*) has high effect on the number of symptomatic infected individuals (shown in Figure 3(e)), but less correlated with *R*_0_. The recovery rate (*γ*_2_) has most impact on *R*_0_, indicated that the reduction of contact rate of asymptomatic infectious could consider for effective control the spread of HFMD. Results suggest that decreasing direct transmission from asymptomatic individuals, although they cannot be diagnosed and detected, contributes to the HFMD dynamics. The indirect transmission via free-living viruses in the environment should be concerned in improving hygienic precautions and strategies for controlling HFMD outbreak.

## 4. Discussion and Conclusions

There are many studies on control and preventive HFMD measures. However, there are a few works that study mathematical models with contaminated environmental effects to simulate HFMD data in Thailand. It is suggested that enteroviruses can survive for a long period in suitable conditions [9]. Thus, contaminated environments could be an important factor for HFMD dynamics. In this study, we have proposed and analysed a HFMD model by extending the existing model for HFMD endemic in Bangkok 2016, including contaminated environmental effects and a subgroup of asymptomatic individuals. We constructed the hospitalized compartment to fit with the HFMD reported data of Bangkok. So that this model can demonstrate rationality of HFMD dynamics, since the most reported data is derived from hospitals. In our analysis, we used R software, which is a free programming language usable for everyone. Our main objectives in studying the HFMD endemic were how indirect transmission and numerous asymptomatic individuals influence the disease dynamics and how the progression rate to hospital decreases the number of symptomatic individuals.

We provided theoretically the basic reproduction number, which is estimated as 1.441 from fitting parameters. This value is greater than the estimation in 2012 [32], in which indirect transmission was neglected. This may contribute to the effect of contaminated environments which could increase the risk of infection because susceptible individuals could pick the virus up outside the host. Moreover, the basic reproduction number was estimated based on the situation of HFMD in 2012. In the case of neglected transmission from asymptomatic individuals (*β*_2_ = 0), the basic reproduction number is less than one. The results indicate that the asymptomatic subgroup also plays an important role in the spread of HFMD which corresponds to the results of [16, 17]. Although, asymptomatic individuals could not be diagnosed, quarantined, and tracked to implement measured control, but they should not be neglected.

Numerical results show that direct transmission by asymptomatic infected individuals and indirect transmission via free-living viruses in contaminated environments are important factors which contribute to new HFMD infections. The progression rate to hospital if symptomatic individuals admitted to hospital immediately is able to decrease the number of symptomatic individuals due to decreasing the chance of direct contact.

Our sensitivity analysis results indicated that the basic reproduction number is sensitive to asymptomatic and symptomatic subgroups parameters (*γ*_2_, *β*_2_, *β*_1_, and *δ*) and indirect transmission via free-living viruses (*ν*, *ϕ*_1_, and *μ*). An effective control strategy could be suggested by reducing the direct transmission contact such as wearing a mask to prevent the spread of viruses. Healthcare education such as washing hands before meals and after using the toilet, making air fresh indoors and so on, should be provided [15]. Contaminated environments could represent an important factor contribute to HFMD dynamics. Cleaning the environment frequently and healthcare precautions should be suggested to be more effective control of the spread of HFMD.

Note that our proposed model is used to fit data from Bangkok, 2016, and estimate some unknown parameters using the genetic algorithm. Seasonal variation is not considered in our model due to climatic data limits. These factors could help us to improve the model since it showed the correlation of HFMD incidence and climatic factors [33]. We assumed that homogeneous mixing of the population, which in Bangkok, is heterogenetic in community structure which could influence transmission dynamics. However, this study focused on the effects of indirect transmission and asymptomatic infected individuals. Our model could be fitted to the reported data of Bangkok 2016, which is an updated incidence data. Furthermore, we used hospitalized compartment in fitting processes, which corresponds to reported data. Thus, the estimated symptomatic infected individuals in our model could provide public health with more information.

In conclusion, our findings suggest that indirect transmission via free-living viruses in contaminated environments and asymptomatic infected individuals play important roles for HFMD dynamics. The reduction of direct and indirect contact to infected individuals could help to improve control strategies. The sensitivity analysis of the basic reproduction number could also help to develop effective intervention strategies, which can optimize healthcare and control measures. Further study should be done by access the effect of community structure or seasonal infection.

## Figures and Tables

**Table 1:** Definitions of parameters in HFMD model.

Parameter	Definitions (unit)	Value	References
*b*	Birth rate (/day)	252	Estimated
*d*	The natural death rate and the rate at which children become adults	0.000267	Assumption
*β*_1_	The transmission rate between *S* and *I*	0.229	fitting
*β*_2_	The transmission rate between *S* and *I*_*e*_	0.492	fitting
1/*σ*	The average incubation period (day)	2.75	fitting
*δ*	The rate of progression to hospitalized individuals (/day)	0.749	fitting
*p*	The fraction of those becoming infectious with symptoms	0.421	fitting
1 − *q*	The fraction of infectious individuals (*I*) being hospitalized (*H*)	0.967	fitting
*ε*	The recovery rate of infectious patients with symptoms (/day)	0.021	fitting
*γ*_1_	The recovery rate of hospitalized individuals (/day)	0.782	fitting
*γ*_2_	The recovery rate of infectious without symptoms (/day)	0.318	fitting
*η*	The rate from recovered to again susceptible (/day)	1/100	[14]
*ϕ*_1_	The virus shedding from infectious individuals with symptoms (/day)	457.504	fitting
*ϕ*_2_	The virus shedding from infectious individuals without symptoms (/day)	22.443	fitting
*ν*	The indirect transmission rate (/day)	1.0 × 10^−6^	fitting
*μ*	The clearance pathogen rate (/day)	698.077	fitting

**Table 2:** PRCC values on the outcome of *R*_0_.

Parameters	Distribution	PRCC	Standard error
*β*_1_	U[0.1 0.3]	0.6280	0.0160
*β*_2_	U[0.4 0.6]	0.9370	0.0031
*σ*	U[0.3 0.5]	0.0204	0.0235
*δ*	U[0.7 0.8]	−0.3952	0.0212
*ε*	U[0.01 0.03]	−0.0211	0.0232
*γ*_2_	U[0.2 0.4]	−0.9821	0.0009
*ϕ*_1_	U[400 500]	0.4698	0.0209
*ϕ*_2_	U[20 25]	0.0894	0.0227
*ν*	U[0.0000009 0.0000011]	0.4927	0.0197
*μ*	U[650 750]	−0.3680	0.0213

## Data Availability

The data used to support the findings of this study are available from the corresponding author upon request.

## Supplementary Materials

Supplementary Materials The basic reproduction number *R*_0_.
