# Supplementary material for: The Modelling of Hand, Foot, and Mouth Disease in Contaminated Environments in Bangkok, Thailand
Source: Comput Math Methods Med. 2018 Jun 3;2018:5168931. doi: 10.1155/2018/5168931 (PMC6008700; doi:10.1155/2018/5168931)
Supplement: Supplementary Materials — The basic reproduction number R0. [file 5168931.f1.docx]

**Supplementary Materials:** The basic reproduction number $R_{0}$

The basic reproduction number $R_{0}$ of the model is calculated by using next generation method approach in [[21](#_ENREF_21), [22](#_ENREF_22)]. For this purpose, we set the right hand of the HFMD model (1) to zero as $f-\upsilon$ with

$f=(\frac{\beta_{1}SI+\beta_{2}SI_{e}}{N} , 0 , 0 , 0 , 0 ,0 )^{T}$, and

$$\upsilon= \left(\begin{matrix} (\sigma+d)E \\ -\sigma pE+\delta(1-q)I+(\varepsilon q+d)I \\ -\sigma(1-p)E+(\gamma_{2}+d)I_{e} \\ -\delta(1-q)I+(\gamma_{1}+d)H \\ -\varepsilon qI-\gamma_{2}I_{e}-\gamma_{1}H+(\eta+d)R \\ -\varphi_{1}I-\varphi_{2}I_{e}+\mu W \end{matrix}\right).$$

The disease free-equilibrium (DFE) of the model (1) is $P^{0}=(S,E,I,I_{e},H,R,W)=(\frac{b}{d},0,0,0,0,0,0).$

We can form the next generation matrix $FV^{-1}$ at disease free-equilibrium, where

$F=\left[\begin{matrix} \begin{matrix} 0 \\ 0 \\ 0 \\ 0 \\ 0 \\ 0 \end{matrix} & \begin{matrix} \beta_{1} \\ 0 \\ 0 \\ 0 \\ 0 \\ 0 \end{matrix} & \begin{matrix} \beta_{2} \\ 0 \\ 0 \\ 0 \\ 0 \\ 0 \end{matrix} & \begin{matrix} \begin{matrix} 0 \\ 0 \\ 0 \\ 0 \\ 0 \\ 0 \end{matrix} & \begin{matrix} 0 \\ 0 \\ 0 \\ 0 \\ 0 \\ 0 \end{matrix} & \begin{matrix} \frac{b\nu}{d} \\ 0 \\ 0 \\ 0 \\ 0 \\ 0 \end{matrix} \end{matrix} \end{matrix}\right]$and

$$V=\left[\begin{matrix} \begin{matrix} c_{1} \\ -p\sigma\\ -(-1-p)\sigma\\ 0 \\ 0 \\ 0 \end{matrix} & \begin{matrix} 0 \\ c_{2} \\ 0 \\ -(1-q)\delta\\ -\varepsilon q \\ -\varphi_{1} \end{matrix} & \begin{matrix} \begin{matrix} 0 \\ 0 \\ c_{3} \\ 0 \\ -\gamma_{2} \\ -\varphi_{2} \end{matrix} & \begin{matrix} 0 \\ 0 \\ 0 \\ d+\gamma_{1} \\ -\gamma_{1} \\ 0 \end{matrix} & \begin{matrix} \begin{matrix} 0 \\ 0 \\ 0 \\ 0 \\ d+\eta\\ 0 \end{matrix} & \begin{matrix} 0 \\ 0 \\ 0 \\ 0 \\ 0 \\ \mu\end{matrix} \end{matrix} \end{matrix} \end{matrix}\right].$$

Where the constants are $c_{1} = d+\sigma$, $c_{2 }= d+(1-q)\delta+\varepsilon q$, and $c_{3} = d+\gamma_{2}$.

Hence, the basic reproduction number of $R_{0}$ is the spectral radius (dominant eigenvalue) of the matrix$FV^{-1}$, namely,

$R_{0}=\frac{p\sigma(b\nu\varphi_{1}+d\mu\beta_{1})}{c_{1}c_{2}d\mu}+\frac{(1-p)(b\sigma\nu\varphi_{2}+d\sigma\mu\beta_{2})}{c_{1}c_{3}d\mu}$.
